# Berberine Inhibits MDA-MB-231 Cells by Attenuating Their Inflammatory Responses

**DOI:** 10.1155/2020/3617514

**Published:** 2020-03-16

**Authors:** Lina Zhao, Chunhai Zhang

**Affiliations:** Department of Thyroid Surgery, China-Japan Union Hospital of Jilin University, Changchun, Jilin Province, China

## Abstract

Breast cancer initiation is closely associated with cytokines that can change the inflammatory tumor microenvironment. Compounds extracted from plants have been explored for the possibility of cancer treatment in the recent decades. Berberine is an isoquinoline plant alkaloid with remarkable antioxidant and anti-inflammation roles, which is used in ethnic medicines, including traditional Chinese and North American medicine. In the present study, we investigated the effects of berberine on the malignant tumor cell behaviors in a breast cell line, MDA-MB-231. We found that berberine could not influence the cell viability in normal condition but was able to decrease the cancer cell migration capability in a scratch wound model and accordingly prolong the wound healing time. Furthermore, our results demonstrated that berberine inhibited the increased phosphorylation of c-Jun and c-Fos in these scratched cancer cells. With the cotreatment with LPS, which could boost the expression of cytokines in these cancer cells, berberine significantly reduced the increased expression of TNF-*α* and IL-6. Meanwhile, we found that berberine inhibited the activation of NF-*κ*B by preventing the degradation of I*κ*B*α*.

## 1. Introduction

Breast cancer is among the major causes of cancer mortality in the female population worldwide [1]. It accounts for one-third of newly diagnosed cancer in women of the United States [2]. Multiple factors are associated with the occurrence of breast cancer, such as body weight or obesity, diet, ethnicities, and gynecology and obstetrical history. Despite the remarkable progress in the treatment of breast cancer surgically and chemotherapeutically, breast cancer still remains one of the key vicious diseases for women in modern society globally. Also, although scientists have put intensive efforts in this field to reveal the molecular mechanisms of breast cancer, the tumorigenesis of breast cancer on the level of pathophysiological conditions is not well profiled yet. There are emerging evidences implying that activation of certain immune receptors in cancer cells could result in the products of proinflammatory cytokines and chemokines in the tumor microenvironment. The increased inflammatory context further initiates the tumorigenesis [3]. Inflammatory condition also facilitates metastasis. Therefore, regulating the inflammatory response in the tumor microenvironment may amend the current treatment regime of breast cancer.

A great deal of studies has revealed that natural compounds extracted from plants may exert anticancer property [4, 5]. Berberine is an isoquinoline alkaloid derived from herbal materials and has been prescribed for diabetes and infections as a complementary treatment in China [6]. Berberine was found to regulate the LPS-induced inflammatory response in macrophages [7]. In breast cancer, berberine could inhibit cancer cell proliferation and cell migration [8]. Moreover, a recent study reported that in a human triple-negative breast cancer cell line, berberine inhibits not only the tumor outgrowth and metastasis in TNBC but also the NLRP3 inflammasome, which leads to markedly reducing a series of cytokine products, including IL-1, IL-6, and TNF-*α* [9]. Therefore, berberine may be a potential therapeutic compound for the breast cancer treatment by regulating the inflammatory microenvironment of cancer cells.

In the present study, we used a human breast cancer cell line, MDA-MB-231, to further elaborate the possible ways that berberine affects the inflammation of cancer cells and the cell behaviors. Our data will provide more information to validate the application of berberine in the treatment of breast cancer.

## 2. Materials and Methods

### 2.1. Cells and Treatment

The human breast cell line MDA-MB-231 is maintained in 35 mm dishes with Dulbecco's Modified Eagle's Medium (DMEM) containing 10% Fetal Bovine Serum (FBS). The cultures were incubated at 37°C in a 5% CO_2_ atmosphere. A scratch wound was made by using the pipette tip in the dishes followed by changing the medium to remove debris. Berberine chloride was purchased from Sigma-Aldrich and dissolved in dimethyl sulfoxide (DMSO) as stock solution. LPS (10 *μ*g/L) was added to the cultures for the inflammation study, and berberine was employed 30 min before the addition of LPS. In a scratch model, berberine was added after changing the medium. Photos were taken 24 hours later after scratching, and the wound healing process was recorded and compared among groups.

### 2.2. Cell Viability

We used the 3-(4,5-dimethylthiazol-2-yl)-2,5-diphenyltetrazolium bromide (MTT) assay to examine the cell viability. Generally, breast cancer cells were plated in 96-well plates, and we added MTT into each well at the final concentration of 10% and incubated them for 4 hours in an incubator. Optical densities were probed at 570 nm, and viabilities were expressed as a relative percentage compared to their controls.

### 2.3. Western Blot

Cultured cancer cells were washed with cold PBS for 3 times, and protein extraction buffer was added to the dishes. With a cell scraper, we collected the total protein and then boiled them for denaturation. The equal amount of total protein was loaded and separated in SDS-PAGE protein gel after determining the protein concentration. The protein was then transferred to a PVDF membrane (Millipore) and blocked with 5% bovine serum albumin (BSA). Primary antibodies of c-jun (Cell Signaling), c-fos (Cell Signaling), NF-*κ*B p65 (Cell Signaling), I*κ*B*α* (Cell Signaling), and *β*-actin (Santa Cruz) were incubated with the membranes at cold room temperature overnight, respectively. HRP-conjugated secondary antibodies were used for further 1 hr incubation at room temperature after washing the membranes with TBST. The corresponding bands were developed and visualized by chemiluminescence with ECL solution. The protein expression was presented as a ratio compared to internal control protein (*β*-actin).

### 2.4. Scratch Wound Assay

A scratch wound model was then created by using plastic pipette tips (10–20 *μ*L pipette tips) as previously reported by [6]. We washed the dishes immediately after scratching to remove dead cells and other debris generated during scratching. The scratched dishes were then treated with or without berberine. During this time, the wound gaps between groups were repeatedly imaged to measure the distance between two edges of the wounds in each dish.

### 2.5. ELISA

The ELISA assay was performed with kits of TNF-*α* and IL-6 (Abcam) according to the protocol provided by the manufacturer. MDA-MB-231 cells were washed with cold PBS, and then, lysis buffer was added to extract the total protein of cells. After protein concentration was determined, the same amount of protein was loaded in the well in a duplicate manner. The expressed levels of TNF-*α* and IL-6 in the cell lysis were normalized with total loading protein.

### 2.6. Statistical Analysis

All values in the present study were expressed as mean ± SEM. Statistical analysis was performed with Prism 5 software. Multiple-group analysis was done with one-way ANOVA followed by the Newman–Keuls post hoc test. Two-group analysis was done with the *t*-test. A *p* value of less than 0.05 was considered statistically significantly different.

## 3. Results

### 3.1. Berberine Had No Effects on the Cancer Cell Viability

**Figure 1:**
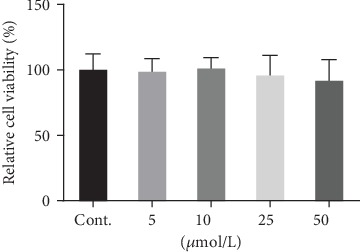
The effects of berberine on the cancer cell viability (24 hours). Data was expressed as mean ± SEM. *n* = 7.

To test the possible anticancer effects of berberine in these breast cancer cells, we first incubated MDA-MB-231 cells with berberine in a range of concentration (Figure 1) for 24 hr. The doses of the berberine used here were based on the previous report from Sarna et al. [7]. As shown in this figure, berberine failed to show any sign of reduction of the viability of these breast cancer cells even in the highest concentration. These results indicated that berberine might not affect the cell viability in certain condition with these above doses. 25 *μ*mol/L was used for the following experiments based the above results and previous report [7].

### 3.2. Berberine Inhibited the Wound Healing Process of the Scratched Breast Cancer Cells

**Figure 2:**
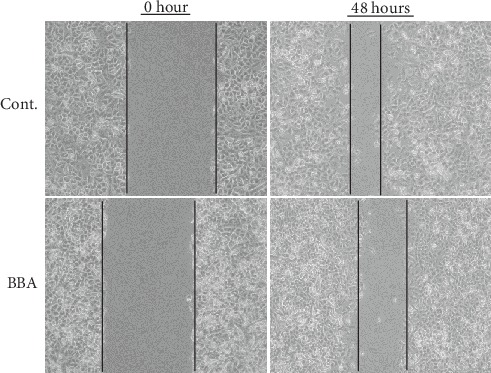
The effects of berberine on the cancer cell migration in a scratch wound model (48 hours). BBA: berberine (25 *μ*mol/L).

Next, we investigated whether berberine could affect the capacity of migration of these cancer cells. We scratched the confluent monolayer cancer cells and monitored the cell migration along the wound edge by photographing the wound in a manner of time course. We found that the cells along the edge started the migration almost immediately after scratching with or without the treatment with berberine. But berberine treatment reduced the migration capacity since the wound gap was much bigger in berberine-treated cells than in the cells without treatment (Figure 2). These results implied that berberine could inhibit the migration of breast cancer cells, which is a feature property of malignancy of metastasis.

### 3.3. Berberine Inhibited the Activation of c-fos and c-jun in the Cancer Cells after Scratching

**Figure 3:**
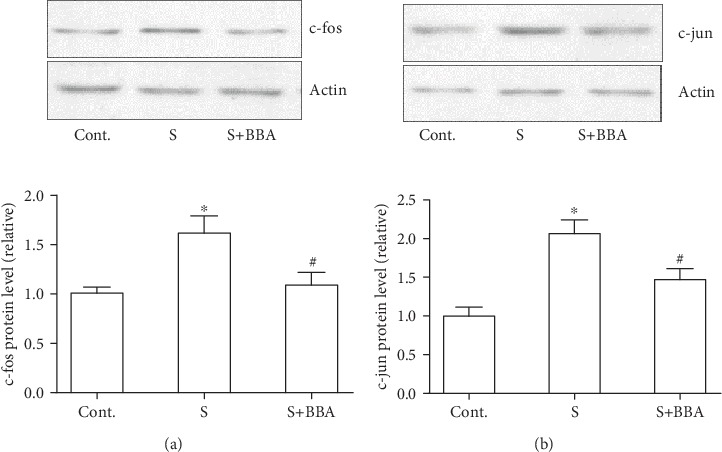
Berberine inhibited the increased expression of c-fos and c-jun in scratched cancer cells. (a) The effect of berberine on the expression of c-fos at 30 min after scratching. (b) The effect of berberine on the expression of c-jun at 30 minutes after scratching. Data was expressed as mean ± SEM. ^∗^*p* < 0.05 compared to control; ^#^*p* < 0.05 compared to scratch (*n* = 5). BBA: berberine (25 *μ*mol/L); S: scratch.

We also tried to identify the involved molecular candidates which might trigger the migration after scratching for the purpose of exploring the possible target proteins of berberine. A study has suggested that early response genes were activated in scratched cells [10]. Therefore, we performed western blot to study the expression level of c-fos and c-jun in these scratched breast cancer cells in the early stage of postscratching (30 minutes). We found that the protein levels of both c-fos and c-jun were boosted after scratching but their expression level inhibited with the cotreatment with berberine (Figures 3(a) and 3(b)).

### 3.4. Berberine Prevented the LPS-Induced Cytokine Expression

**Figure 4:**
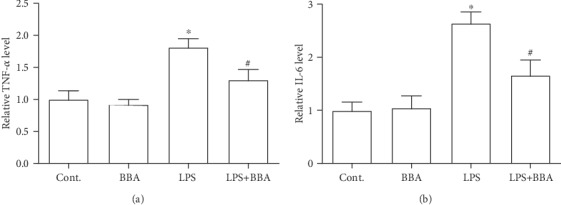
Berberine inhibited the increased expression of TNF-*α* and IL-6 in cancer cells exposed to LPS. (a) The effect of berberine on the expression of TNF-*α* at 6 hours after LPS treatment. (b) The effect of berberine on the expression of IL-6 at 6 hours after LPS treatment. Data was expressed as mean ± SEM. ^∗^*p* < 0.05 compared to control; ^#^*p* < 0.05 compared to LPS (*n* = 7). BBA: berberine (25 *μ*mol/L).

Breast cancer cells could boost the capacity of generating cytokines [11]. To explore the possible effects of berberine on the inflammation in breast cancer cells, LPS was used to treat these cancer cells in the next experiments. With the studies of ELISA, we found that the products of TNF-*α* and IL-6 in these cells were significantly increased compared to control groups (Figures 4(a) and 4(b)). Interestingly, cotreatment with berberine was able to effectively decrease the expression levels of these two cytokines (Figures 4(a) and 4(b)). These results demonstrated that berberine might exert anti-inflammation effects on the breast cancer cells.

### 3.5. Berberine Prevented the Increased Expression of NF-*κ*B p65 and the Degradation of I*κ*B*α*

**Figure 5:**
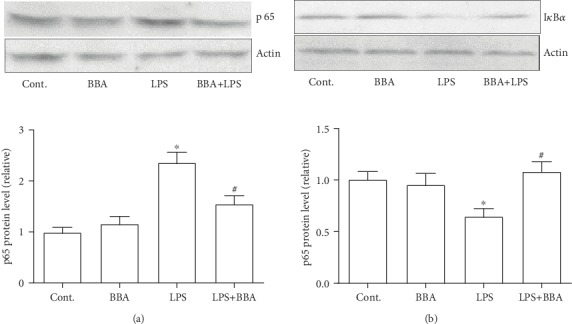
Berberine inhibited the increased expression of NF-*κ*B p65 and prevented the protein loss of I*κ*B*α* in cancer cells exposed to LPS. (a) The effect of berberine on the expression of NF-*κ*B p65 at 6 hours after LPS treatment. (b) The effect of berberine on the expression of I*κ*B*α* at 6 hours after LPS treatment. Data was expressed as mean ± SEM. ^∗^*p* < 0.05 compared to control; ^#^*p* < 0.05 compared to LPS (*n* = 6). BBA: berberine (25 *μ*mol/L).

Last, we investigated the possible mechanism by which berberine exerted those beneficial effects on breast cancer cells. We performed western blot to measure the protein level of NF-*κ*B p65. We found an increased expression level of p65 in cancer cells exposed to LPS, but this increase could be prevented by berberine (Figure 5(a)). Accordingly, there was reducing expression of I*κ*B*α* that is the inhibiting protein of NF-*κ*B in the LPS-treated cancer cells. However, the cotreatment with berberine improved the expression level of I*κ*B*α* (Figure 5(b)). The above data implicated that berberine might reduce the inflammatory response in breast cancer cells by increasing the expression of I*κ*B*α*, which then led to inhibiting the p65 protein.

## 4. Discussion

Diagnosis, care, and prognosis of breast cancer have improved significantly during the past decades with the further understanding of their pathogenies and etiology. However, breast cancer is still a major threat to female in middle age, especially in economically developed countries, and represents one of the top biomedical research priorities globally [12]. Although it remains still unknown, extensive efforts have been devoted to find the initiation process of breast cancer. Inflammatory response in the microenvironment was considered an active player in the initiation, promotion, angiogenesis, and metastasis [13]. Consequently, the boosted expression of cytokine, activated cytokine receptor, and intracellular signaling of NF-*κ*B facilitated the malignant cell behaviors [14]. Therefore, a compound which could target the inflammatory reaction in the tumor microenvironment as a supplementary treatment to the mainstream chemotherapy could influence the outcome and further optimize the treatment regime of breast cancer.

Compounds derived from natural plants have been extensively studied of their application in many fields, including cancers. As mentioned above, berberine was found to possess prominent features in anti-inflammation and antioxidant function. Here, we used this compound to test several hypotheses in the breast cancer cell line. Our data here were in line with previous reports [15]. Furthermore, we found that berberine could inhibit the breast cell migration in a scratch wound injury. Since increased migration capacity is a feature of malignant cell behavior in cancer cells, the role of berberine in the cell migration is a foundation finding in this study. And based on the finding, we explored the possible mechanism in several ways, such as inflammation-related signaling pathway and early response genes. The c-fos and c-jun signaling pathway has been studied for many years of their role in the cell migration [16, 17]. In the present study, we found that scratching caused increased protein expression levels of these two proteins, which could be inhibited by the berberine treatment. These results implied that inhibiting the c-fos and c-jun protein response in the early stage might be one of the possible mechanisms. It has been well established that the NF-*κ*B signaling pathway is the center of inflammation reaction [17, 18]. In the study, we found that berberine inhibits the expression of p65 which was triggered by the LPS stimulation in breast cancer cells. More importantly, we also revealed that the inhibiting component of NF-*κ*B and I*κ*B*α* was regulated by the berberine treatment. As shown in Figure 5, the expression level of I*κ*B*α* was significantly decreased in LPS-treated cancer cells, which indicated a desuppression of NF-*κ*B. With the cotreatment with berberine, the degradation of I*κ*B*α* was prevented and resulted in reduced expression level of p65, accordingly. A previous study also indicated that berberine could inhibit the phosphorylation of NF-*κ*B and c-fos and c-jun proteins and lung cell invasion [19]. In the present study, we found that berberine inhibited the increased expression levels of c-fos and c-jun and the cell migration in the scratch wound model. Combined with the results of the NF-*κ*B signaling pathway mentioned above, we concluded that berberine might affect the NF-*κ*B signaling pathway and exert its beneficial effects on cancer cell migration and inflammatory response.

In a nutshell, our study provided more evidences to support the application of berberine in the treatment of breast cancer cells at cellular and molecular levels. To further confirm the role of berberine, an animal study is warranted for the future.

## Data Availability

The data used to support the findings of this study are available from the corresponding author upon request.
